# Inositol-requiring enzyme 1 alpha is essential for dentinogenesis

**DOI:** 10.3389/fphys.2025.1722417

**Published:** 2025-12-08

**Authors:** Qian Xu, Tian Liang, Jiahe Li, Suzhen Wang, Hua Zhang, Julie Hollien, Takao Iwawaki, Chunlin Qin, Yongbo Lu

**Affiliations:** 1 Department of Biomedical Sciences, Texas A&M University College of Dentistry, Dallas, TX, United States; 2 Eastman Institute for Oral Health, School of Medicine and Dentistry, University of Rochester, Rochester, NY, United States; 3 Department of Orthodontics and Pediatric Dentistry, University of Michigan School of Dentistry, Ann Arbor, MI, United States; 4 School of Biological Sciences and Center for Cell and Genome Science, University of Utah, Salt Lake City, UT, United States; 5 Department of Life Science, Medical Research Institute, Kanazawa Medical University, Ishikawa, Japan

**Keywords:** inositol-requiring enzyme 1 alpha (IRE1α), dentin sialophosphoprotein (DSPP), odontoblast, dentin formation, unfolded protein response (UPR), dentinogenesis imperfecta (DGI)

## Abstract

**Introduction:**

Inositol-requiring enzyme 1 alpha (IRE1α), encoded by endoplasmic reticulum (ER) to nucleus signaling 1 (*Ern1*) gene, is the most conserved sensor of ER stress. IRE1α-initiated signaling pathways contribute to functional maturation of secretory cells and have been implicated in various human diseases. In this study, we examined the roles of IRE1α in odontoblast development and dentin formation in wild-type mice as well as in *Dspp* ^*P19L*^ mutant mice, which express a pathogenic variant of dentin sialophosphoprotein (P19L-DSPP) and exhibit a dentinogenesis imperfecta (DGI)-like phenotype.

**Methods:**

Western-blotting and stains-all staining analyses were used to assess whether secretion of mutant P19L-DSPP was impaired in dental pulp cells containing odontoblasts from *Dspp* ^*P19L/P19L*^ mice compared with *Dspp* ^*+/+*^ controls. Immunohistochemistry and reverse-transcription PCR were performed to examine changes in IRE1α and its downstream target X-box binding protein 1 (XBP1) in P19L-DSPP mutant mice. To further investigate the roles of IRE1α in tooth development, we generated *2.3 Col1-Cre;Ern1* ^*fl/fl*^ and compound *2.3 Col1-Cre;Ern1* ^*fl/fl*^;*Dspp* ^*P19L/+*^ mice. Structural and histological changes in mandibular molars were analyzed using plain X-ray radiography, micro-computed tomography (µCT), and histology. Additionally, *in situ* hybridization, quantitative real-time PCR, and immunohistochemistry were performed to compare molecular changes among these mice and *Ern1* ^*fl/fl*^ and *Ern1* ^*fl/fl*^;*Dspp* ^*P19L/+*^ controls.

**Results:**

Western-blotting and stains-all staining analyses support that mutant P19L-DSPP protein was not efficiently secreted into dentin matrix and was accumulated within odontoblasts. Further, immunostaining signals for phosphorylated IRE1α and total XBP1 were dramatically increased in odontoblasts and other dental pulp cells of *Dspp* ^*P19L/+*^ and *Dspp* ^*P19L/P19L*^ mice, in comparison with *Dspp* ^*+/+*^ mice. Consistently, there was a small increase in spliced XBP1S protein and *Xbp1s* mRNA levels in P19L-DSPP mutant mice. Moreover, loss of IRE1α function reduced dentin formation in 2.3 *Col1-Cre;Ern1* ^*fl/fl*^ mice and exacerbated the dental defects of P19L-DSPP mutant mice. Notably, IRE1α deficiency did not restore the *Dspp* mRNA levels in the mutant mice but normalized the increased thickness of the dental pulp chamber floor dentin.

**Conclusion:**

These findings underscore the essential role of IRE1α in odontoblast function and dentinogenesis. Moreover, they reveal a context-dependent pathogenic role of IRE1α, providing new insights into ER stress in dental tissue development and disease.

## 1 Introduction

The endoplasmic reticulum (ER) is an intracellular organelle within eukaryotic cells. It is the entry point for proteins destined to enter the secretory pathway and responsible for folding and processing secretory and transmembrane proteins; it ensures that only properly folded proteins are allowed to exit the ER, while the misfolded/unfolded proteins are retained in the ER and targeted for ER-associated degradation (Ruggiano et al., 2014). The ER maintains a balance between the unfolded proteins that enter the ER and the folding and exporting capacity of the ER, a condition known as “ER homeostasis.” Any physiological or pathological disturbance to this homeostasis may result in an accumulation of misfolded/unfolded proteins called “ER stress”. In response to stress, the ER activates three major signaling pathways that are respectively initiated by three ER transmembrane sensors, inositol-requiring enzyme 1 alpha (IRE1α), protein kinase RNA-like endoplasmic reticulum kinase (PERK), and activating transcription factor 6 (ATF6), which are collectively named the “unfolded protein response (UPR)” (Ron and Walter, 2007; Walter and Ron, 2011). The UPR functions to restore ER homeostasis.

IRE1α is encoded by the *Ern1* gene and is the most conserved sensor of ER stress (Tirasophon et al., 1998; Wang et al., 1998). It is a Type I transmembrane protein, consisting of an N-terminal ER luminal domain, a transmembrane domain, and a cytoplasmic serine/threonine kinase and endoribonuclease domain (Mori et al., 1993; Tirasophon et al., 1998; Miyoshi et al., 2000). Under unstressed conditions, IRE1α exists in its inactive monomer state (Bertolotti et al., 2000). Upon ER stress, IRE1α undergoes dimerization and trans-autophosphorylation, leading to activation of its endoribonuclease domain (Kimata et al., 2007; Ron and Hubbard, 2008; Korennykh et al., 2009; Oikawa et al., 2009; Li et al., 2010; Tam et al., 2014). The activated IRE1α endoribonuclease catalyzes a frameshift splicing of the mRNA encoding unspliced X-box binding protein 1 (XBP1U) to produce a spliced XBP1 mRNA that encodes spliced XBP1 (XBP1S) (Yoshida et al., 2001; Calfon et al., 2002; Lee et al., 2002). XBP1U undergoes rapid degradation after synthesis, whereas XBP1S is a potent transcription factor that enters the nucleus and induces the transcription of target genes to promote protein folding and degradation of misfolded proteins present in the ER (Yoshida et al., 2001; Calfon et al., 2002; Tirosh et al., 2006; Yoshida et al., 2006; Navon et al., 2010). IRE1α′s endoribonuclease activity can also degrade other mRNAs localized to the ER membrane through a process known as regulated IRE1-dependent decay (RIDD), down-regulating their translation and potentially reducing the amount of nascent proteins that enter the ER to alleviate ER stress (Hollien and Weissman, 2006; Han et al., 2009; Hollien et al., 2009; Nakamura et al., 2011; Gaddam et al., 2013; Coelho and Domingos, 2014; Tam et al., 2014; Moore and Hollien, 2015). IRE1α-XBP1S signaling is indispensable for functional maturation of various types of secretory cells, such as pancreatic acinar cells, salivary gland acinar cells, gastric zymogenic cells, plasma cells, hepatocytes and osteoblasts (Reimold et al., 2000; Reimold et al., 2001; Gass et al., 2002; Iwakoshi et al., 2003; Shaffer et al., 2004; Lee et al., 2005; Huh et al., 2010; Tohmonda et al., 2011). In addition, IRE1α has been implicated in various human diseases, including cancer, diabetes, inflammatory diseases, neurodegenerative disorders, liver and cardiovascular diseases (Hetz et al., 2011; Luo et al., 2022; Wang et al., 2022; Wang et al., 2023; Shi et al., 2024; Tak et al., 2025; Zhou et al., 2025).

Accumulating evidence suggests that ER stress and the UPR may also be involved in inherited dental defects caused by mutations in the gene encoding dentin sialophosphoprotein (DSPP). DSPP is a non-collagenous extracellular matrix protein (Fisher et al., 2001; Fisher and Fedarko, 2003). It is continuously secreted by odontoblasts, but only transiently produced by differentiating ameloblasts during tooth development (D'Souza et al., 1997; Ritchie et al., 1997; Begue-Kirn et al., 1998; MacDougall et al., 1998; Bleicher et al., 1999). DSPP is synthesized as a single large protein, which is proteolytically cleaved into an N-terminal fragment called dentin sialoprotein (DSP) and a C-terminal fragment known as dentin phosphoprotein (DPP) (MacDougall et al., 1997; Sun et al., 2010; von Marschall and Fisher, 2010; Zhu et al., 2012). DSP is a proteoglycan containing two glycosaminoglycan chains (Ritchie et al., 1994; Zhu et al., 2010; Yamakoshi et al., 2011), whereas DPP is a highly phosphorylated and acidic protein (Butler et al., 1983; George et al., 1996; Ritchie and Wang, 1996). Mutations in the *DSPP* gene in humans cause a non-syndromic inheritable dominant dental disorder, known as dentinogenesis imperfecta (DGI) (Shields et al., 1973; MacDougall et al., 2006; Kim and Simmer, 2007; Barron et al., 2008; McKnight et al., 2008; Nieminen et al., 2011; Li et al., 2012). We previously generated a *Dspp* ^*P19L*^ mouse model that expressed a mutant DSPP in which the proline residue at position 19 (P19) was replaced by a leucine residue (p.P19L) (Liang et al., 2019). This mouse model is equivalent to one of 5′ *DSPP* human mutations, c.50C>T, which results in the substitution of proline residue at position 17 with a leucine residue (p.P17L) (Li et al., 2012; Lee et al., 2013; Porntaveetus et al., 2019). Indeed, we reported that both *Dspp* ^*P19L/+*^ and *Dspp* ^*P19L/P19L*^ mice developed a DGI-like phenotype, resembling the phenotype of human patients carrying the corresponding p. P17L mutation (Li et al., 2012; Lee et al., 2013; Porntaveetus et al., 2019). In addition, we showed that the P19L-DSPP mutant mice had hypoplastic enamel, delayed enamel maturation as well as ultrastructural enamel defects (Liang et al., 2021). Such dentin and enamel defects were associated with an accumulation of the mutant DSPP protein within odontoblasts and presecretory ameloblasts (Liang et al., 2019; Liang et al., 2021). Our previous studies also demonstrated that the secretion of P19L-DSPP was impaired and the mutant P19L-DSPP was accumulated within the ER of the expressing cells (Liang et al., 2019; Liang et al., 2023). An accumulation of the mutant DSPP protein in the ER may disrupt ER homeostasis and cause ER stress. Accordingly, we have found that the levels of *Dspp* mRNAs were dramatically reduced in both *Dspp* ^*P19L/+*^ and *Dspp* ^*P19L/P19L*^ mice (Liang et al., 2019), suggesting that RIDD might be activated in these mutant mice.

In this study, we first examined if IRE1α was activated in the odontoblasts in *Dspp* ^*P19L/+*^ and *Dspp* ^*P19L/P19L*^ mice, compared to wild-type mice. We then generated *2.3 Col1-Cre;Ern1* ^*fl/fl*^ and compound *2.3 Col1-Cre;Ern1* ^*fl/fl*^ *;Dspp* ^*P19L/+*^ mice to delete the *Ern1* gene in the odontoblasts to investigate the roles of IRE1α in dentinogenesis in wild-type mice and in *Dspp* ^*P19L/+*^ mice, respectively. We observed that IRE1α-XBP1S signaling was minimally activated in the odontoblasts in the P19L-DSPP mutant mice. We demonstrated that loss of IRE1α function reduced dentin formation in *2.3 Col1-Cre;Ern1* ^*fl/fl*^ mice. Further, IRE1α inactivation worsened the dental defects and failed to restore the *Dspp* mRNA levels in *2.3 Col1-Cre;Ern1* ^*fl/fl*^ *;Dspp* ^*P19L/+*^ mice. Notably, IRE1α deficiency restored the thickened dental pulp chamber floor dentin to normal in *2.3 Col1-Cre;Ern1* ^*fl/fl*^ *;Dspp* ^*P19L/+*^ mice. These findings not only support that IRE1α is essential for odontoblast function and dentin formation, but also highlight the context-dependent pathogenic role of IRE1α in the P19L-DSPP mutant mice.

## 2 Materials and methods

### 2.1 Animals

All mice used in this study were maintained on a C57BL/6 background and were bred and maintained in community housing (≤4 mice/cage, 22 °C) on a 12-h light/dark cycle with free access to water and standard pelleted food. All animal procedures were approved by the Institutional Animal Care and Use Committee (IACUC) of Texas A&M University.

### 2.2 Generation of *Dspp* ^*P19L/+*^, *Dspp* ^*P19L/P19L*^, *2.3 Col1-Cre;Ern1* ^*fl/fl*^ and *2.3 col1-Cre;Ern1* ^*fl/fl*^ *;Dspp* ^*P19L/+*^ mice

*Dspp* ^*P19L/+*^ and *Dspp* ^*P19L/P19L*^ mice express a mutant DSPP, in which the proline residue at position 19 was replaced by a leucine residue (p.P19L), and their generation was described in our previous report (Liang et al., 2019). IRE1α is encoded by the *Ern1* gene that is ubiquitously expressed in mammals (Miyoshi et al., 2000). Conventional inactivation of *Ern1* gene leads to early embryonic lethality at E12.5 (Urano et al., 2000; Zhang et al., 2005; Iwawaki et al., 2009). To determine the roles of IRE1α in *Dspp*-mutant mice, *Ern1* floxed (*Ern1* ^*fl/+*^) mice were first bred with *2.3-kb Col1a1-Cre* (*2.3 Col1-Cre*) transgenic mice to generate *2.3 Col1-Cre;Ern1* ^*fl/fl*^ mice. The *Ern1* ^*fl/+*^ mice carried an *Ern1* allele with exons 20–21 flanked by two loxP recombination sites (Iwawaki et al., 2009). The *2.3-kb Col1a1-Cre* transgenic mice express a Cre recombinase driven by a 2.3-kb mouse type I collagen alpha 1 chain (Col1a1) promoter, which is active in odontoblasts in tooth and in osteoblasts in bone (Dacquin et al., 2002). The *Ern1* ^*fl/+*^ mice were also mated with the *Dspp* ^*P19L/+*^ mice to generate *Ern1* ^*fl/fl*^ *;Dspp* ^*P19L/+*^ mice. *2.3 Col1-Cre;Ern1* ^*fl/fl*^ mice were then crossed with *Ern1* ^*fl/fl*^ *;Dspp* ^*P19L/+*^ mice to generate *Ern1* ^*fl/fl*^, *2.3 Col1-Cre;Ern1* ^*fl/fl*^, *Ern1* ^*fl/fl*^ *;Dspp* ^*P19L/+*^ and *2.3 Col1-Cre;Ern1* ^*fl/fl*^ *;Dspp* ^*P19L/+*^. *Ern1* ^*fl/fl*^ mice were used as normal control mice; *2.3 Col1-Cre;Ern1* ^*fl/fl*^ mice, in which *Ern1* gene was deleted in the odontoblasts, were used to investigate the role of IRE1α in dentinogenesis in the presence of normal *Dspp* gene; *Ern1* ^*fl/fl*^ *;Dspp* ^*P19L/+*^ mice were used as the *Dspp*-mutant control mice; and *2.3 Col1-Cre;Ern1* ^*fl/fl*^ *;Dspp* ^*P19L/+*^ mice, in which *Ern1* was ablated in the odontoblasts in *Dspp* ^*P19L/+*^ genetic background, were used to investigate the roles of *IRE1α* in *Dspp*-mutant mice. Mice of all the genotypes studied were fertile and bred normally. Both male and female mice were used for analyses, as there were no phenotypic differences between sexes for each genotype.

### 2.3 Protein extraction, western-blotting analysis and stains-all staining

The mandibular incisors were extracted from 3-month-old *Dspp* ^*+/+*^ and *Dspp* ^*P19L/P19L*^ mice and were immediately frozen by liquid nitrogen until further processing. The four mandibular incisors from two mice for each genotype were combined and ground into powder in a precooled mortar with pestle. The powder was transferred into a sterile 1.5 mL Eppendorf (EP) tube containing 6 M urea supplemented with protease inhibitor (1 tablet/10 mL; Roche, Basel, Switzerland). The dental pulps/odontoblasts were resuspended and then separated from the dentin/enamel powder by centrifugation (any floating tissues are dental pulp tissues), and were transferred into a new EP tube for extraction of the proteins from the dental pulp/odontoblasts. Six molar urea containing 0.5 M EDTA and protease inhibitor was then added to the precipitated dentin/enamel powder to extract the dentin matrix proteins. The extracted proteins were concentrated using Amicon Ultra- 0.5 mL (Cat# UFC501096), and protein concentration was determined by DS-11 Spectrophotometer (DeNovix, Wilmington, DE).

For Western-blotting analysis, 10 or 20 μg of the total proteins extracted from dental pulp/odontoblasts and dentin matrices were electrophoresed on 4%–15% gradient SDS-PAGE (sodium dodecyl sulfate-polyacrylamide gel electrophoresis) gels (BioRad, Hercules, CA), which were then transferred onto a PVDF membrane (EMD Millipore, Billerica, CA). Membranes were blocked in 5% milk (Labscientific, Highlands, NJ) in tris-buffered saline with 0.1% Tween-20 detergent (TBST), and immunoblotted with a rabbit anti-DSP polyclonal antibody (recognizing both DSP and full-length DSPP, 1:4000) (Qin et al., 2003a), followed by incubation with HRP-conjugated goat anti-rabbit IgG antibody (1:2000; Santa Cruz Biotechnology, Inc., Dallas, TX). The immunostained protein bands were detected with ECL™ Chemiluminescent detection reagents (Pierce Biotechnology, Inc., Rockford, IL) and imaged by a CL-XPosure film (Pierce Biotechnology, Inc., Rockford, IL).

Stains-all staining was performed to visualize all the acidic non-collagenous proteins including DSPP and its processed DSP and DPP fragments, as previously described (Sun et al., 2010; Zhu et al., 2012). Briefly, 10, 20 or 60 μg of the total proteins extracted from dental pulp/odontoblasts and dentin matrices were electrophoresed on 4%–15% gradient SDS-PAGE gels (BioRad), which were then stained using Stains-all (Sigma, Saint Louis, MO).

### 2.4 RNA extraction, reverse-transcription PCR and quantitative real-time PCR (qPCR)

Total RNAs were extracted from the dental pulps of the first molars of 3-week-old *Dspp* ^*+/+*^, *Dspp* ^*P19L/+*^, and *Dspp* ^*P19L/P19L*^ mice as well as 3-week-old *Ern1* ^*fl/fl*^, *2.3 Col1-Cre;Ern1* ^*fl/fl*^, *Ern1* ^*fl/fl*^ *;Dspp* ^*P19L/+*^ and *2.3 Col1-Cre;Ern1* ^*fl/fl*^ *;Dspp* ^*P19L/+*^ mice, as previously described (Liang et al., 2019). Briefly, both the maxillary and mandibular first molars were extracted from each mouse and were combined as one sample. The four first molars were ground into powder in a mortar with pestle precooled by liquid nitrogen, and total RNAs were extracted using TRIzol Reagent (Invitrogen, Waltham, MA), according to the manufacturer’s instruction.

For reverse-transcription PCR analysis of *Xbp1* mRNAs, 0.5 µg of total RNAs from each sample were reverse transcribed into cDNAs using QuantiTect Reverse Transcription Kit (Qiagen, Germantown, MD), according to the manufacturer’s instruction. The *Xbp1* cDNAs were then amplified using the following primers: forward primer 5′-GAACCAGGAGTTAAGAACACG-3′ and reverse primer 5′-AGGCAACAGTGTCAGAGTCC-3′, as previously described (Iwawaki et al., 2004). The plasmid containing spliced *Xbp1* cDNA (pFLAG.XBP1p.CMV2; Addgene, Watertown, MA) and the plasmid containing unspliced *Xbp1* cDNA (pFLAG.XBP1u.CMV2; Addgene, Watertown, MA) were a gift from David Ron (Calfon et al., 2002), and were used as the controls. The PCR products were resolved by electrophoresis on a 3% agarose gel. The gels were imaged with Azure C150 gel imaging system (Azure Biosystems, Dublin, CA), and the density of each PCR band was quantified using ImageJ program (Schneider et al., 2012). The amplicons from *Dspp* ^*+/+*^, *Dspp* ^*P19L/+*^, and *Dspp* ^*P19L/P19L*^ mice were subjected to enzymatic digestion by PstI. The percent of the *XBP1S* amplicon in each sample was calculated based on the densities of the PCR bands corresponding to this equation: (Hx0.5 + S)/(H + S + U1+U2). Four independent samples were analyzed for each genotype. The quantified data shown represented mean ± SD.

For quantitative real-time PCR (qPCR) analysis, 1 µg of total RNAs from each sample were reverse transcribed into cDNAs using QuantiTect Reverse Transcription Kit (Qiagen), according to the manufacturer’s instruction. The resulting cDNAs were then diluted in a ratio of 1 to 4 using RNase-free water for qPCR analyses of *Dspp* and dentin matrix protein 1 (*Dmp1*) mRNAs, as previously described (Liang et al., 2019). Glyceraldehyde-3-phosphate dehydrogenase (*Gapdh*) was used as the internal control. Briefly, qPCR was performed using GoTaq qPCR Master Mix (Promega, Madison, WI), according to the manufacturer’s instruction. The qPCR reaction was set as 95 °C for 3 min as an initial denaturation, followed by 40 cycles of (95 °C for 30 s, 60 °C for 60 s, and plate read), then 72 °C for 7 min. Table 1 showed the primers used in this study. A BioRad CFX96 Touch Real-time PCR Detection System with its built-in software was used for qPCR. The data obtained from 5 independent mice for each group were used for the quantitative analysis.

**TABLE 1:** Primers used for quantitative PCR.

Target	Forward primer	Reverse primer
*Dspp*	5′-CTGGGAAGAGCCAAGATCAG-3′	5′-GTCAGACTCCCCTTGCTTTG-3′
*Dmp1*	5′-AGTGAGTCATCAGAAGAAAGTCAAGC-3′	5′-CTATACTGGCCTCTGTCGTAGCC-3′
*Gapdh*	5′-CTCCTGGAAGATGGTGATGG-3′	5′-GGCAAAGTGGAGATTGTTGC-3′

### 2.5 Plain X-ray radiography and micro-computed tomography (μCT)

The mandibles were dissected from 3-week-old *Dspp* ^*+/+*^, *Dspp* ^*P19L/+*^, and *Dspp* ^*P19L/P19L*^ mice as well as 3- and 7-week-old *Ern1* ^*fl/fl*^, *2.3 Col1-Cre;Ern1* ^*fl/fl*^, *Ern1* ^*fl/fl*^ *;Dspp* ^*P19L/+*^ and *2.3 Col1-Cre;Ern1* ^*fl/fl*^ *;Dspp* ^*P19L/+*^ mice and were fixed in 4% paraformaldehyde (PFA) in diethylpyrocarbonate (DEPC)-treated 0.1 M phosphate-buffered saline (PBS) overnight. The left halves of the mandibles were then stored in 70% ethanol until further analysis. For plain X-ray radiography, the left halves of the mandibles were imaged with a high-resolution Faxitron X-Ray MX-20 Specimen Radiography System (Faxitron X-Ray Corp., Tucson, AZ) at 6s/26 kV for 3-week-old mice and at 10.6s/26 kV for 7-week-old mice. The left halves of the mandibles were then scanned with a high-resolution Scanco μCT35 imaging system (Scanco Medical, Brüttisellen, Switzerland) with a slice increment of 6 μm at 70 kV and 114 μA, as previously described (Gibson et al., 2013; Liang et al., 2019; Chavez et al., 2021). For three-dimensional (3D) structure construction and morphometric analysis of the mandibular first molars, the whole teeth were outlined. Thresholds were determined for each age based on visual comparisons (Christiansen, 2016), that could distinguish the tissue of interest from the surrounding tissues. For enamel, a threshold of 550 was used for 3-week-old mice, while a threshold of 580 was used for 7-week-old mice. For dentin and cementum together, a threshold of 250 and 270 were chosen for 3- and 7-week-old mice, respectively. The morphometric parameters, including the volume and density, were evaluated using the μCT built-in software. For measuring roof and floor dentin thickness, the lowest point at the upper border of the roof dentin concave and the highest point at the lower border of the floor dentin convex were taken as reference points. The roof dentin thickness and floor dentin thickness were defined as the thickness of dentin on the line determined by the two reference points on the sagittal plane that transverses the center of the mandibular first molars. The center of the mandibular first molar was defined as the sagittal (mesial to distal) section crossing both the most proximal and distal pulp horns, which usually bring two more pulp horns between them, and with the largest openings of both proximal and distal root apexes. The central 10 slices were measured for the roof dentin thickness and floor dentin thickness for each mouse. An average of 10 measurements were taken as the thickness of roof dentin and floor dentin, respectively, for each mouse. The data obtained from 5 independent mice for each group were used for the quantitative analysis.

### 2.6 Sample processing and histological analysis

The right halves of the dissected mouse mandibles were harvested and fixed in 4% paraformaldehyde (PFA) in DEPC-treated 0.1 M PBS overnight. The mandibles were then decalcified in 15% ethylenediaminetetraacetic acid (EDTA) solution (pH 7.4) at 4 °C for 7–14 days, based on the age. The decalcified mandibles were then dehydrated in gradient ethanol (50% ethanol for 1 h, 70% ethanol for 1 h, 95% ethanol for 2 h and 100% ethanol for 1 h twice and then overnight) and xylene (for 1 h twice), and were embedded in paraffin subsequently, and were cut into a series of mesio-distal sections at a thickness of 5 μm for hematoxylin and eosin (H&E) staining and other histological analyses, as previously described (Gibson et al., 2013; Liang et al., 2019; Liang et al., 2021).

### 2.7 Immunohistochemistry (IHC)

Immunohistochemistry (IHC) was performed as previously described (Gibson et al., 2013; Liang et al., 2019). Briefly, the 5-µm sections were processed in xylene and gradient ethanol for dewax and rehydration, were then incubated sequentially in sodium citrate buffer (pH 6.0) for antigen retrieval and in PBS containing 3% hydrogen peroxidase (H_2_O_2_) for quenching endogenous peroxidase. The sections were then blocked with 3% bovine serum albumin (BSA) and 10% normal goat serum (NGS) in 0.1 M PBST (0.1M PBS with 0.1% Tween-20), followed by sequential incubation with primary and secondary antibodies diluted in 2% NGS. The primary antibodies used in this study included: 1) rabbit anti-phosphorylated IRE1α polyclonal antibody (1:400, Novus Biologicals, NB100-2323); 2) rabbit anti-XBP1 polyclonal antibody that recognizes both unspliced XBP1 (XBP1U) and spliced XBP1 (XBP1S) (1:200, Abcam, ab37152); 3) rabbit anti-XBP1S monoclonal antibody (E9V3E) that specifically recognizes XBP1S (Xu et al., 2021; Xu et al., 2023) (1:50, Cell Signaling Technology, Danvers, MA); 4) rabbit anti-DSPP polyclonal antibody that recognizes both DSP and full-length DSPP (1:2000) (Qin et al., 2003a); and 5) rabbit anti-DMP1 polyclonal antibody (1:800, #857) (Qin et al., 2003b). The biotinylated goat anti-rabbit IgG (H + L) antibody (1:200, Vector Laboratories, Burlingame, CA) was use as the secondary antibody. The immunostaining signals were visualized using DAB (3.3′-diaminobenzidine) kit (Vector Laboratories, Burlingame, CA), according to the manufacturer’s instruction. The sections were counterstained with methyl green (Sigma, Saint Louis, MO) for better visualization of the tissue morphology and were then mounted with Permount mounting medium (Fisher Scientific, Waltham, MA). Images were taken with a Leica DM4 B Automated Upright Microscope System (Leica Biosystems, Wetzlar, Germany). IHC analyses were performed on tissue sections from three mice per genotype to assess each protein of interest.

IHC staining for XBP1S was quantified using ImageJ software (NIH, USA). DAB-stained images were processed by color deconvolution to isolate the DAB channel. The odontoblast and pulp cell layers were manually defined as regions of interest (ROIs), including floor-forming odontoblasts, roof-forming odontoblasts, and pulp cells. A consistent threshold was applied to each ROI to identify positively stained areas. The percentage of DAB-positive area relative to the total tissue area within each ROI was calculated. Three representative areas for each cell population were analyzed per sample, and the mean values were used for statistical comparison between groups.

### 2.8 *In situ* hybridization (ISH)

2.8

*In situ* hybridization (ISH) was carried out to detect *Dspp* and *Dmp1* transcripts using a digoxigenin (DIG)–labeled antisense complementary RNA (cRNA) probe, as previously described (Gibson et al., 2013; Liang et al., 2019). Briefly, following deparaffinization and rehydration, tissue sections were permeabilized by 10 μg/mL protease K (Ambion, Austin, TX) for 5 min at room temperature, and were then hybridized with 1 μg/mL DIG-labeled 1.1 kb antisense cRNA probe for mouse *Dspp* transcripts or 0.8 kb antisense cRNA probe for mouse *Dmp1* transcripts at 65 °C for 14–16 h. The sections were blocked, and immunostained with an anti-DIG antibody conjugated to alkaline phosphatase (1:2000, Roche, Basel, Switzerland) and developed with an NBT/BCIP (nitro blue tetrazolium/5-bromo-4-chloro-3-indolyl-phosphate) chromogenic substrate system (Roche, Basel, Switzerland). The sections were then counterstained with nuclear fast red (Sigma, Saint Louis, MO) and mounted with Permount mounting medium (Fisher Scientific, Waltham, MA). Images were taken with a Leica DM4 B Automated Upright Microscope System (Leica Biosystems). ISH analyses were conducted on tissue sections from three mice per genotype to analyze *Dspp* and *Dmp1* mRNA expression.

### 2.9 Statistical analysis

Statistical analyses were conducted using the GraphPad Prism 9.0 software package (GraphPad Software, San Diego, CA). One-way analysis of variance (ANOVA) was conducted to compare the differences among three or four groups. If significant differences were found by one-way ANOVA, the Tukey test was used as *post hoc* test. The quantified results were represented as mean ± standard deviation (SD). *p* < 0.05 was considered statistically significant.

## 3 Results

### 3.1 An accumulation of mutant P19L-DSPP protein within the odontoblasts of *Dspp* ^*P19L/P19L*^ mice

**FIGURE 1:**
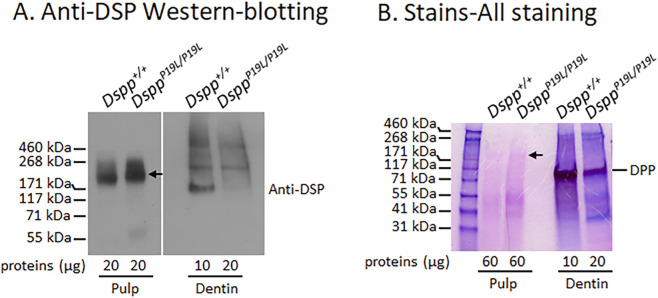
Analyses of mutant P19L-DSPP protein in the dental pulp and dentin matrix. Shown are the representative anti-DSP Western-blotting analysis **(A)** and Stains-all staining **(B)** of total proteins extracted from the dental pulps and dentin matrices of 3-month-old *Dspp* ^*+/+*^ and *Dspp* ^*P19L/P19L*^ mouse incisors. Arrows indicate the full-length DSPP protein. Please note that the amount of dentin matrix proteins loaded from *Dspp* ^*P19L/P19L*^ mice was twice as much as that from *Dspp* ^*+/+*^ mice. Two independent experiments for both anti-DSP Western-blotting analysis and Stains-all staining yielded similar results.

We have previously reported that both molars and incisors were affected in *Dspp* ^*P19L/+*^ and *Dspp* ^*P19L/P19L*^ mice, accompanied by an intracellular accumulation and endoplasmic reticulum retention of mutant P19L-DSPP in the odontoblasts and presecretory ameloblasts (Liang et al., 2019; Liang et al., 2021; Liang et al., 2023). Mouse mandibular incisors are large and continuously grow and erupt, providing a source of actively secreting odontoblasts. Therefore, we extracted total proteins from the dental pulps (containing odontoblasts) and dentin matrices of 3-month-old *Dspp* ^*P19L/P19L*^ mouse mandibular incisors, and then analyzed the full-length mutant P19L-DSPP protein and its processed fragments in both preparations by anti-DSP Western-blotting (Figure 1A) and Stains-all staining (Figure 1B). Anti-DSP Western-blotting analysis was performed using a rabbit anti-DSP polyclonal antibody that recognizes both DSP and full-length DSPP (Sun et al., 2010; Liang et al., 2019), whereas Stains-all staining shows DSPP and its processed DPP as the blue-stained protein bands (Sun et al., 2010; Zhu et al., 2012). Anti-DSP Western-blotting analysis demonstrated that the dental pulps of the *Dspp* ^*P19L/P19L*^ mice contained more full-length DSPP protein than those of the wild-type *Dspp* ^+*/*+^ control mice (Figure 1A, pulp), which was confirmed by Stains-all staining (Figure 1B, pulp). In contrast, both anti-DSP Western-blotting and Stains-all staining analyses showed that the dentin matrices from the *Dspp* ^*P19L/P19L*^ mice contained much less DSP/DSPP-related proteins, including DPP that can be only revealed by Stains-all staining, than those from the *Dspp* ^+*/*+^ mice (Figures 1A,B, Dentin). It is of particular note that the amount of dentin matrix proteins from *Dspp* ^*P19L/P19L*^ mice was loaded twice as much as that from *Dspp* ^*+/+*^ mice, indicating that the dentin matrices from the *Dspp* ^*P19L/P19L*^ mice had at least two folds less DPP proteins than those of the *Dspp* ^+*/*+^ mice. These results further supported that the mutant P19L-DSPP protein was not efficiently secreted into the dentin matrix, and was accumulated within the odontoblasts.

### 3.2 IRE1α-XBP1S signaling was weakly activated in the dental pulp cells of the P19L-DSPP mutant mice

**FIGURE 2:**
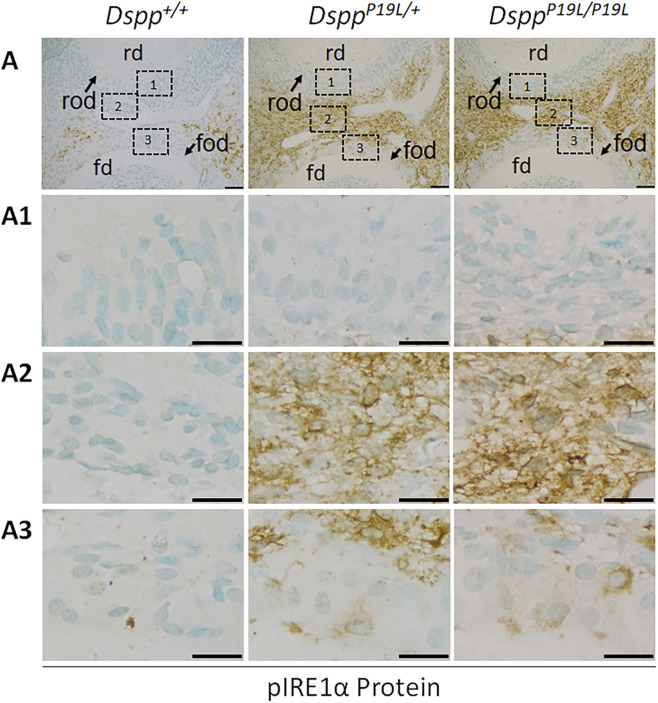
Immunohistochemistry staining of phosphorylated IRE1α (pIRE1α) protein in the mandibular first molars. (A) Shown are the representative images of immunohistochemistry staining of pIRE1α protein (signal in brown) in the mandibular first molars of 3-week-old *Dspp* ^*+/+*^, *Dspp* ^*P19L/+*^, and *Dspp* ^*P19L/P19L*^ mice. Each image in **(A)** is from the middle region of the crown of a sagittally-sectioned mandibular first molar. (A1-A3) are the higher magnification views of the roof-forming odontoblasts (box1), dental pulp cells (box 2) and floor-forming odontoblasts (box 3) in the corresponding images in **(A)**, respectively. rd, roof dentin; fd, floor dentin; rod, roof-forming odontoblasts; fod, floor-forming odontoblasts; Scale bars: 50 μm in A; 20 μm in (A1-A3). Three independent experiments for IHC staining of pIRE1α show similar results.

**FIGURE 3:**
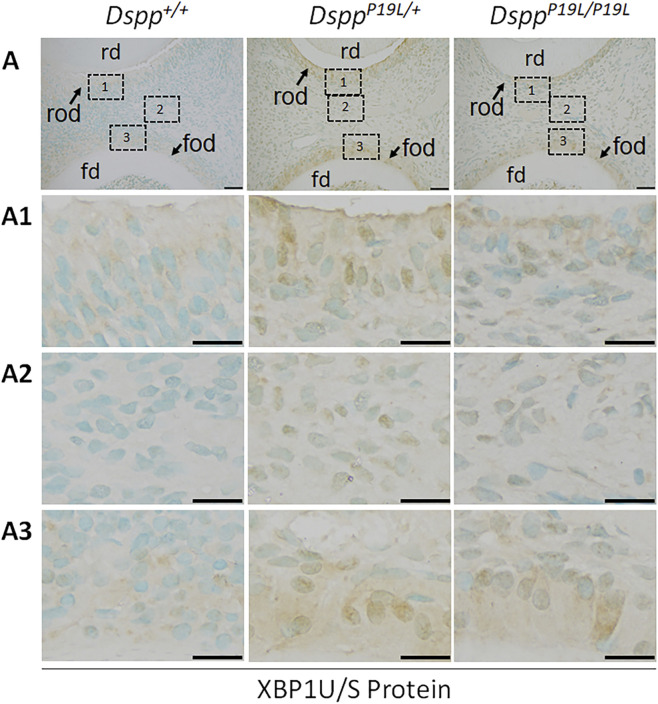
Immunohistochemistry staining of total XBP1 protein in the mandibular first molars. (A) Shown are the representative images of immunohistochemistry staining of total XBP1 (including XBP1U and XBP1S) protein (signal in brown) in the mandibular first molars of 3-week-old *Dspp* ^*+/+*^, *Dspp* ^*P19L/+*^, and *Dspp* ^*P19L/P19L*^ mice. Each image in **(A)** is from the middle region of the crown of a sagittally-sectioned mandibular first molar. (A1-A3) are the higher magnification views of the roof-forming odontoblasts (box1), dental pulp cells (box 2) and floor-forming odontoblasts (box 3) in the corresponding images in **(A)**, respectively. rd, roof dentin; fd, floor dentin; rod, roof-forming odontoblasts; fod, floor-forming odontoblasts. Scale bars: 50 μm in A; 20 μm in (A1-A3). Three independent experiments for IHC staining of total XBP1 produce similar results.

**FIGURE 4:**
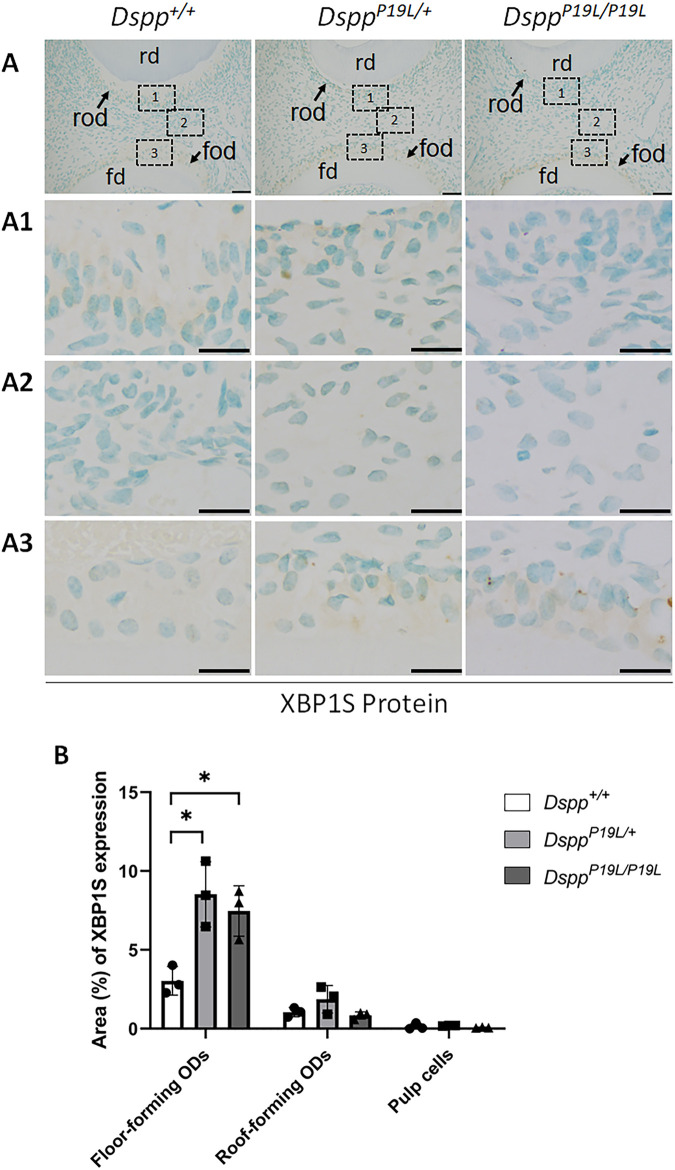
Immunohistochemistry staining of spliced XBP1 (XBP1S) protein in the mandibular first molars. **(A)** Shown are the representative images of immunohistochemistry staining of XBP1S protein (signal in brown) in the mandibular first molars of 3-week-old *Dspp* ^*+/+*^, *Dspp* ^*P19L/+*^, and *Dspp* ^*P19L/P19L*^ mice. Each image in **(A)** is from the middle region of the crown of a sagittally-sectioned mandibular first molar. (A1-A3) are the higher magnification views of the roof-forming odontoblasts (box1), dental pulp cells (box 2) and floor-forming odontoblasts (box 3) in the corresponding images in **(A)**, respectively. **(B)** Quantification of XBP1S immunohistochemistry staining. The percentage of DAB-positive area in roof-forming odontoblasts, floor-forming odontoblasts, and dental pulp cells was measured using ImageJ. Data are shown as mean ± SD. *, *p* < 0.05. rd, roof dentin; fd, floor dentin; rod, roof-forming odontoblasts; fod, floor-forming odontoblasts. ODs, odontoblasts. Scale bars: 50 μm in **(A)**; 20 μm in (A1-A3). Three independent experiments were performed for IHC staining of XBP1S.

**FIGURE 5:**
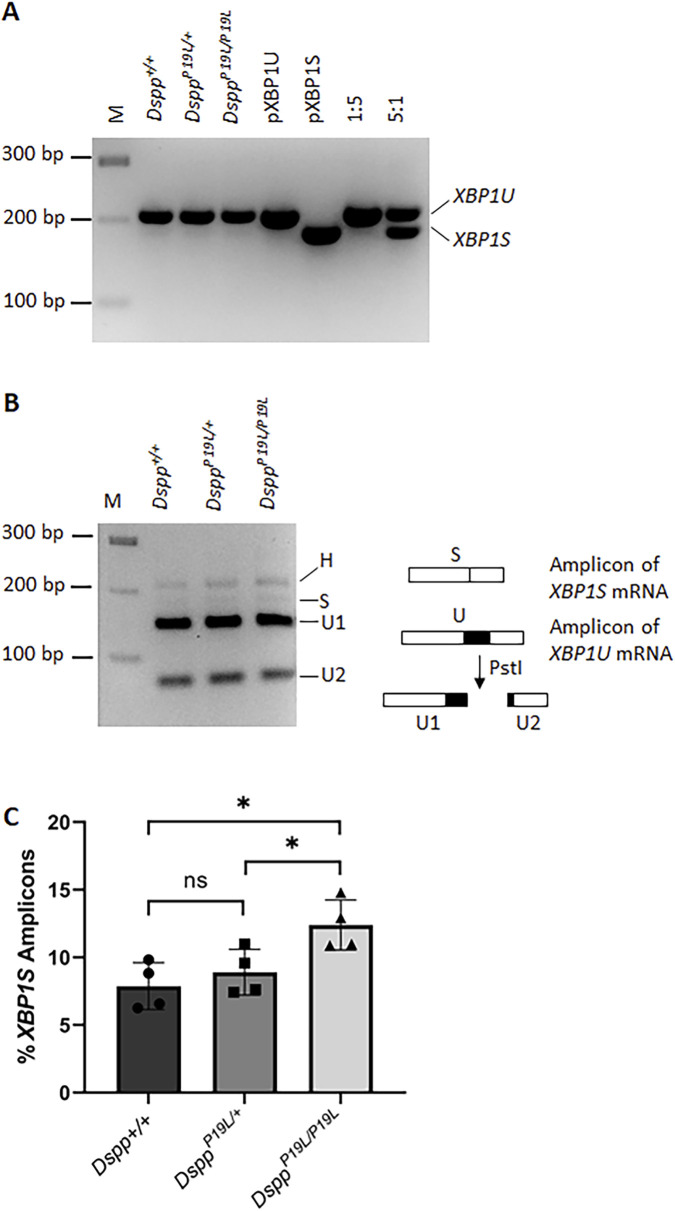
RT-PCR analysis of unspliced *Xbp1* (*Xbp1u*) and spliced *Xbp1* (*Xbp1s*) mRNAs in the dental pulps. **(A)** RT-PCR was performed to detect the amount of *Xbp1* (including *Xbp1u* and *Xbp1s*) mRNAs in the total RNAs extracted from the dental pulps of the first molars of 3-week-old *Dspp* ^*+/+*^, *Dspp* ^*P19L/+*^, and *Dspp* ^*P19L/P19L*^ mice. PCR products corresponding to *Xbp1u* mRNA and *Xbp1s* mRNA are indicated. pXBP1U, unspliced *Xbp1* plasmid control; pXBP1S, spliced *Xbp1* plasmid control; 1:5, the ratio of pXBP1S plasmid to pXBP1U plasmid; and 5:1, the ratio of pXBP1S plasmid to pXBP1U plasmid. **(B)** The amplicons from *Dspp* ^*+/+*^, *Dspp* ^*P19L/+*^, and *Dspp* ^*P19L/P19L*^ mice were subjected to enzymatic digestion by PstI. H, hybrid; M, DNA molecular weight markers. **(C)** The percentage of the *Xbp1s* amplicon in each sample was calculated based on this equation, (Hx0.5 + S)/(H + S + U1+U2). Four independent samples were analyzed for each genotype. Each data point represents the data obtained from one independent mouse. n = 4. *, *p* < 0.05; ns, no significance.

We next examined the IRE1α branch of the UPR to determine if it was activated by the intracellularly accumulated mutant P19L-DSPP in the dental pulps of 3-week-old *Dspp* ^*P19L/+*^ and *Dspp* ^*P19L/P19L*^ mice, compared to the age-matched *Dspp* ^+*/*+^ control mice (Figure 2). IHC with an antibody that detects the phosphorylated IRE1α (pIRE1α) showed that pIRE1α immunostaining signals were remarkably stronger in the odontoblasts (particularly in the floor-forming odontoblasts) and other dental pulp cells in *Dspp* ^*P19L/+*^ and *Dspp* ^*P19L/P19L*^ mice than in *Dspp* ^+*/*+^ mice (Figure 2). We then performed IHC and reverse-transcription PCR (RT-PCR) analyses to determine the protein and mRNA levels of XBP1U (unspliced XBP1) and XBP1S (spliced XBP1), in the odontoblasts and other dental pulp cells of 3-week-old *Dspp* ^*P19L/+*^ and *Dspp* ^*P19L/P19L*^ mice (Figures 3–5). The immunostaining signals for total XBP1 (including XBP1U and XBP1S) were stronger in the nuclei of the odontoblasts and other dental pulp cells of 3-week-old *Dspp* ^*P19L/+*^ mice and *Dspp* ^*P19L/P19L*^ mice, compared to *Dspp* ^*+/+*^ mice (Figure 3). When an antibody that specifically recognizes XBP1S was used (Xu et al., 2021; Xu et al., 2023), the immunostaining signals were fairly weak in general in all three groups of mice (Figure 4). Nevertheless, it is evident that there was a slight increase in the XBP1S immunostaining signals, particularly in the floor-forming odontoblasts, in *Dspp* ^*P19L/+*^ and *Dspp* ^*P19L/P19L*^ mice, compared to *Dspp* ^*+/+*^ mice (Figure 4). Consistently, a combined reverse-transcription polymerase chain reaction (RT-PCR) and enzymatic digestion analysis showed that the level of *Xbp1s* mRNA was increased in the dental pulp of *Dspp* ^*P19L/P19L*^ mice, compared to *Dspp* ^*+/+*^ and *Dspp* ^*P19L/+*^ mice (Figure 5), though the increase was quite small. Collectively, these findings indicate that IRE1α-XBP1S signaling was weakly activated in the dental pulp cells of the mutant P19L-DSPP mice.

### 3.3 Loss of IRE1α function resulted in reduced dentin formation in mice

**FIGURE 6:**
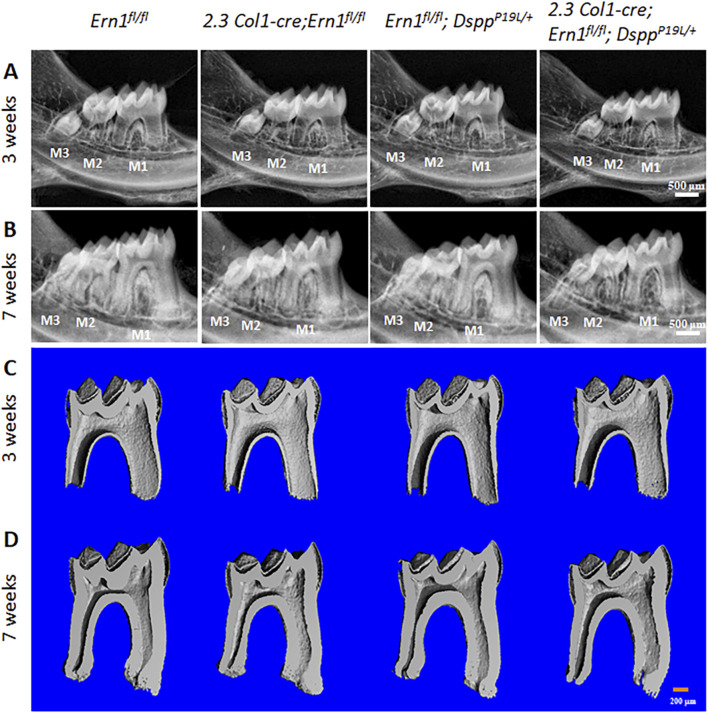
Plain X-ray radiographic and micro-computed tomography (µCT) analyses of the mandibular molars. **(A,B)** Representative plain X-ray radiographs of the mandibular molars of 3-week-old **(A)** and 7-week-old **(B)** *Ern1* ^*fl/fl*^, *2.3 Col1-Cre;Ern1* ^*fl/fl*^, *Ern1* ^*fl/fl*^ *;Dspp* ^*P19L/+*^ and *2.3 Col1-Cre; Ern1* ^*fl/fl*^ *;Dspp* ^*P19L/+*^ mice. M1, first molar; M2, second M; M3, third molar. Scale bars: 500 μm. **(C,D)** Representative 3-dimensional reconstructed μCT images (sagittal sections) of the mandibular first molars of 3-week-old **(C)** and 7-week-old **(D)** *Ern1* ^*fl/fl*^, *2.3 Col1-Cre;Ern1* ^*fl/fl*^, *Ern1* ^*fl/fl*^ *;Dspp* ^*P19L/+*^ and *2.3 Col1-Cre;Ern1* ^*fl/fl*^ *;Dspp* ^*P19L/+*^ mice. The mesial side of each molar is on the right, and the distal side is on the left. Scale bars: 200 μm.

**FIGURE 7:**
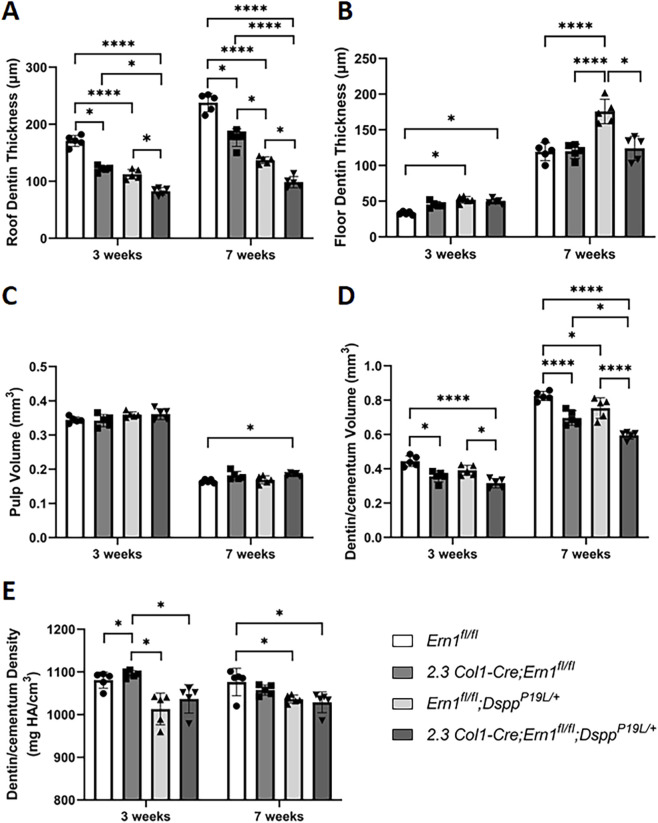
Quantitative µCT analysis of mandibular first molars. Micro-CT analysis was performed to quantify the roof dentin thickness **(A)**, floor dentin thickness **(B)**, pulp volume **(C)**, dentin/cementum volume **(D)** and dentin/cementum density **(E)** of mandibular first molars of 3- and 7-week-old mice. All values are mean ± SD. n = 5 for each group in A-E; *, *p* < 0.05; ****, *p* < 0.0001.

**FIGURE 8:**
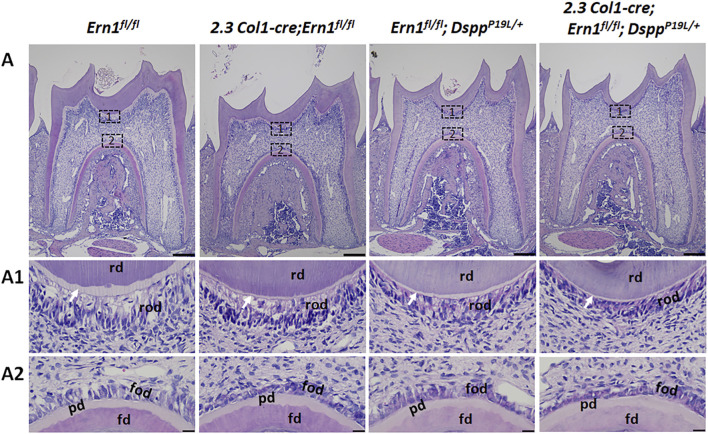
Hematoxylin and eosin (H&E) staining of the mandibular first molars. **(A)** Shown are the representative H&E staining images of the mandibular first molars of 3-week-old *Ern1* ^*fl/fl*^, *2.3 Col1-Cre;Ern1* ^*fl/fl*^, *Ern1* ^*fl/fl*^ *;Dspp* ^*P19L/+*^ and *2.3 Col1-Cre;Ern1* ^*fl/fl*^ *;Dspp* ^*P19L/+*^ mice. The mesial side of each molar is on the right, and the distal side is on the left. (A1-A2) are the higher magnification views of the areas marked box 1 and box 2 in the corresponding images in **(A)**, respectively. Note that the pulp chamber roof predentin (white arrows) became much thinner in *2.3 Col1-Cre;Ern1* ^*fl/fl*^, *Ern1* ^*fl/fl*^ *;Dspp* ^*P19L/+*^ and *2.3 Col1-Cre;Ern1* ^*fl/fl*^ *;Dspp* ^*P19L/+*^ mice, compared to *Ern1* ^*fl/fl*^ mice (A1). Abbreviations: rd, roof dentin; fd, floor dentin; pd, predentin; od, odontoblasts. Scale bars: 200 μm in A; 20 μm in (A1-A2).

We then examined the roles of IRE1α in normal dentinogenesis. To this end, we generated *2.3 Col1-Cre*;*Ern1* ^*fl/fl*^ conditional knockout mice with the *Ern1* gene (encoding IRE1α) specifically deleted in the odontoblasts. We analyzed the effects of the loss of IRE1α function in odontoblasts on tooth development in 3- and 7-week-old *2.3 Col1-Cre*;*Ern1* ^*fl/fl*^ mice, compared to the age-matched *Ern1* ^*fl/fl*^ mice (normal control). Plain X-ray radiographic and 3D reconstructed µCT images showed that *2.3 Col1-Cre*;*Ern1* ^*fl/fl*^ mice had reduced thickness of the dental pulp chamber roof dentin, compared to *Ern1* ^*fl/fl*^ control mice (Figure 6). Quantitative µCT analysis further confirmed that *2.3 Col1-Cre*;*Ern1* ^*fl/fl*^ mice manifested a significant decrease in the thickness of pulp chamber roof dentin at both ages examined when compared to the age-matched *Ern1* ^*fl/fl*^ mice (Figure 7A). However, *2.3 Col1-Cre*;*Ern1* ^*fl/fl*^ mice had increased thickness of pulp chamber floor dentin, though it was not significant when compared to *Ern1* ^*fl/fl*^ control mice at the age of 3 weeks (Figure 7B). By the age of 7 weeks, the thickness of the pulp chamber floor dentin in *2.3 Col1-Cre*;*Ern1* ^*fl/fl*^ mice became comparable to that in *Ern1* ^*fl/fl*^ mice (Figure 7B). Moreover, *2.3 Col1-Cre*;*Ern1* ^*fl/fl*^ mice showed a significant decrease in total dentin/cementum volume at both ages examined when compared to the age-matched *Ern1* ^*fl/fl*^ mice (Figure 7D), but they had a significant increase in dentin/cementum density at the age of 3 weeks (Figure 7E). Histologically, the odontoblasts became shorter in *2.3 Col1-Cre*;*Ern1* ^*fl/fl*^ mice, compared to the long columnar odontoblasts in *Ern1* ^*fl/fl*^ mice (Figure 8). It is of note that the dental pulp chamber roof predentin, but not the pulp chamber floor predentin, became much thinner in *2.3 Col1-Cre*;*Ern1* ^*fl/fl*^ mice than that in *Ern1* ^*fl/fl*^ mice.

**FIGURE 9:**
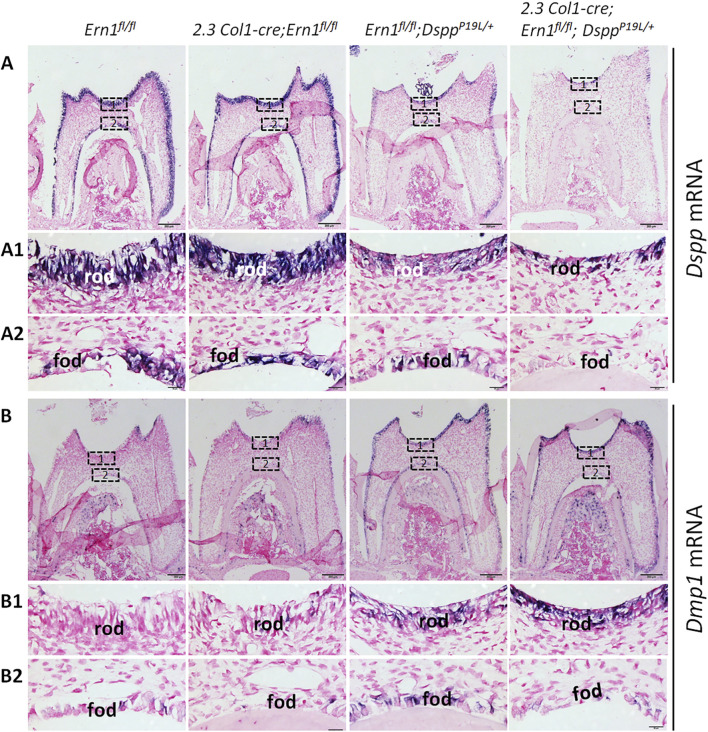
*In situ* hybridization analyses of *Dspp* and *Dmp1* mRNA in the odontoblasts in the mandibular first molars. Shown are the representative *in situ* hybridization analyses (signal in purple) of *Dspp* mRNA **(A)** and *Dmp1* mRNA **(B)** in the mandibular first molars of 3-week-old *Ern1* ^*fl/fl*^, *2.3 Col1-Cre;Ern1* ^*fl/fl*^, *Ern1* ^*fl/fl*^ *;Dspp* ^*P19L/+*^ and *2.3 Col1-Cre; Ern1* ^*fl/fl*^ *;Dspp* ^*P19L/+*^ mice. (A1-A2) are the higher magnification views of the areas marked box1 and box 2 in the corresponding images in **(A)**, respectively. (B1-B2) are the higher magnification views of the areas marked box1 and box 2 in the corresponding images in **(B)**, respectively. rod, roof-forming odontoblasts; fod, floor-forming odontoblasts. Scale bars: 200 μm in A-B; 20 μm in (A1-A2) and (B1-B2). Three independent experiments for ISH analyses of *Dspp* and *Dmp1* mRNA show similar results.

**FIGURE 10:**
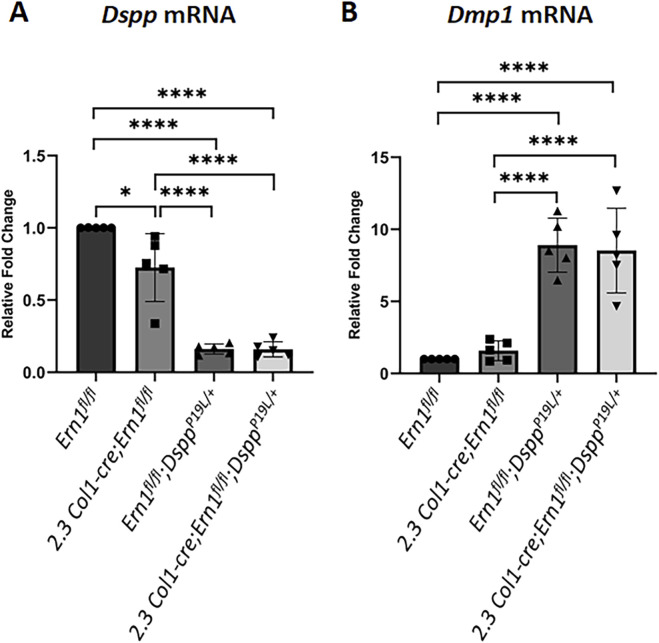
Quantitative real-time polymerase chain reaction (qPCR) analyses of *Dspp* and *Dmp1* mRNA levels in the odontoblasts of the mouse first molars. Shown are qPCR analyses of *Dspp* mRNA **(A)** and *Dmp1* mRNA **(B)** levels in the mouse first molars of 3-week-old *Ern1* ^*fl/fl*^, *2.3 Col1-Cre;Ern1* ^*fl/fl*^, *Ern1* ^*fl/fl*^ *;Dspp* ^*P19L/+*^ and *2.3 Col1-Cre;Ern1* ^*fl/fl*^ *;Dspp* ^*P19L/+*^ mice. The mRNA level of *Ern1* ^*fl/fl*^ mice was set as 1, and the mRNA levels of the *2.3 Col1-Cre;Ern1* ^*fl/fl*^, *Ern1* ^*fl/fl*^ *;Dspp* ^*P19L/+*^ and *2.3 Col1-Cre;Ern1* ^*fl/fl*^ *;Dspp* ^*P19L/+*^ mice were expressed as folds of that in *Ern1* ^*fl/fl*^ mice. *Gapdh* was used as the internal control. The data represent five analyses (n = 5) for each group. Each data point represents the data obtained from one independent mouse. Values are mean ± SD. *, *p* < 0.05; ****, *p* < 0.0001.

**FIGURE 11:**
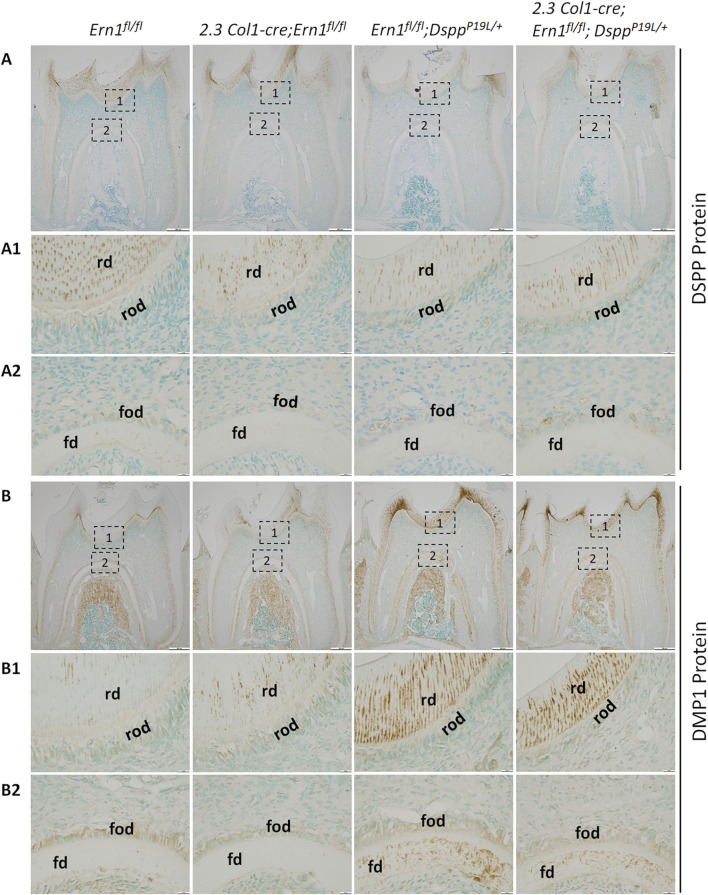
Immunohistochemistry staining of DSP/DSPP and DMP1 protein in the mandibular first molars. Shown are the representative immunohistochemistry staining of DSP/DSPP protein **(A)** and DMP1 protein **(B)** in the mandibular first molars of 3-week-old *Ern1* ^*fl/fl*^, *2.3 Col1-Cre;Ern1* ^*fl/fl*^, *Ern1* ^*fl/fl*^ *;Dspp* ^*P19L/+*^ and *2.3 Col1-Cre;Ern1* ^*fl/fl*^ *;Dspp* ^*P19L/+*^ mice. (A1-A2) are the higher magnification views of the areas marked box1 and box 2 in the corresponding images in **(A)**, respectively. (B1-B2) are the higher magnification views of the areas marked box1 and box 2 in the corresponding images in **(B)**, respectively. rd, roof dentin; rod, roof-forming odontoblasts; fd, floor dentin; fod, floor-forming odontoblasts. Scale bars: 200 μm in **(A,B)**; 20 μm in (A1-A2) and (B1-B2). Three independent experiments for IHC staining of DSP/DSPP and DMP1 show similar results.

We also examined the expression of the *Dspp* and *Dmp1* genes at the mRNA level by *in situ* hybridization (ISH) and quantitative real-time PCR (qPCR) and at the protein level by IHC. DMP1, like DSPP, is a member of the small integrin-binding ligand N-linked glycoprotein family (Fisher et al., 2001), and is essential for dentin formation (Ye et al., 2004). ISH and qPCR analyses showed that there was a slight but significant decrease in *Dspp* mRNA level in *2.3 Col1-Cre*;*Ern1* ^*fl/fl*^ mice, compared to *Ern1* ^*fl/fl*^ mice (Figures 9A,A1–A2; Figure 10A). Yet, IHC showed no obvious difference in the level and distribution of DSP/DSPP-related proteins in the dental pulps and dentin matrices between *2.3 Col1-Cre*;*Ern1* ^*fl/fl*^ mice and *Ern1* ^*fl/fl*^ mice (Figures 11A,A1–A2). Moreover, there were no apparent changes in the levels of *Dmp1* mRNA and DMP1 protein in *2.3 Col1-Cre*;*Ern1* ^*fl/fl*^ mice, compared to *Ern1* ^*fl/fl*^ mice (Figure 9B,B1–B2; Figure 10B; Figures 11B,B1–B2). These data demonstrate that the loss of IRE1α function in the odontoblasts caused reduced dentin formation accompanied by a slight but significant reduction in *Dspp* mRNA level in mice.

### 3.4 Loss of IRE1α function worsened the dental phenotype of P19L-DSPP mutant mice

Meanwhile, we generated compound *2.3 Col1-Cre;Ern1* ^*fl/fl*^ *;Dspp* ^*P19L/+*^ mice to specifically delete the *Ern1* gene in the odontoblasts in *Dspp* ^*P19L/+*^ mice in order to investigate the pathogenic roles of IRE1α in the P19L-DSPP mutant mice. We examined the effects of IRE1α inactivation on tooth development in 3- and 7-week-old *2.3 Col1-Cre;Ern1* ^*fl/fl*^ *;Dspp* ^*P19L/+*^ mice, compared to the age-matched *Ern1* ^*fl/fl*^ *;Dspp* ^*P19L/+*^ mice. We found that *Ern1* ^*fl/fl*^ *;Dspp* ^*P19L/+*^ mice exhibited decreased thickness of dental pulp chamber roof dentin, increased thickness of pulp chamber floor dentin and thinner roof predentin associated with shorter and irregularly arranged odontoblasts, compared to age-matched *Ern1* ^*fl/fl*^ control mice (Figures 6, 7A,B, 8). In addition, they also showed a significant decrease in total dentin/cementum volume and density by 7 weeks, compared to age-matched *Ern1* ^*fl/fl*^ control mice (Figures 7D,E). The *2.3 Col1-Cre;Ern1* ^*fl/fl*^ *;Dspp* ^*P19L/+*^ mice displayed a much thinner dental pulp chamber roof dentin that was even significantly thinner than *Ern1* ^*fl/fl*^ *;Dspp* ^*P19L/+*^ mice at both ages examined (Figures 6, 7A). However, even though the thicknesses of the dental pulp chamber floor dentin of the *2.3 Col1-Cre;Ern1* ^*fl/fl*^ *;Dspp* ^*P19L/+*^ mice were comparable to that of *Ern1* ^*fl/fl*^ *;Dspp* ^*P19L/+*^ mice at the age of 3 weeks, they became significantly reduced, compared to that of *Ern1* ^*fl/fl*^ *;Dspp* ^*P19L/+*^ mice by the age of 7 weeks; and they were reduced to a thickness that was close to that of *Ern1* ^*fl/fl*^ control mice (Figures 6, 7B). Further, *2.3 Col1-Cre;Ern1* ^*fl/fl*^ *;Dspp* ^*P19L/+*^ mice acquired an enlarged dental pulp chamber, compared to *Ern1* ^*fl/fl*^ mice by the age of 7 weeks (Figures 6, 7C). Moreover, *2.3 Col1-Cre;Ern1* ^*fl/fl*^ *;Dspp* ^*P19L/+*^ mice showed a significant reduction in dentin/cementum volume when compared to *Ern1* ^*fl/fl*^ *;Dspp* ^*P19L/+*^ mice (Figure 7D), though the dentin/cementum density was comparable to that of *Ern1* ^*fl/fl*^ *;Dspp* ^*P19L/+*^ mice (Figure 7E). Histologically, *2.3 Col1-Cre;Ern1* ^*fl/fl*^ *;Dspp* ^*P19L/+*^ mice exhibited shortest and most irregularly arranged odontoblasts among the four groups of mice (Figure 8). They also showed a thinner roof predentin, similar to *Ern1* ^*fl/fl*^ *;Dspp* ^*P19L/+*^ mice (Figure 8). Overall, these findings indicate that loss of IRE1α function in the odontoblasts aggravated the dental defects of *Dspp* ^*P19L/+*^ mice, but restored the thickened dental pulp chamber floor dentin to normal.

### 3.5 *Dspp* mRNA levels remained low after IRE1α was inactivated in P19L-DSPP mutant mice

3.5

We also examined the levels of *Dspp* mRNA and DSPP protein in the compound *2.3 Col1-Cre;Ern1* ^*fl/fl*^ *;Dspp* ^*P19L/+*^ mice, compared to those in the age-matched *Ern1* ^*fl/fl*^ *;Dspp* ^*P19L/+*^ mice. ISH demonstrated that *Ern1* ^*fl/fl*^ *;Dspp* ^*P19L/+*^ mice, like *Dspp* ^*P19L/+*^ mice (Liang et al., 2019), had a remarkable decrease in *Dspp* mRNA level in the odontoblasts, compared to *Ern1* ^*fl/fl*^ control mice (Figures 9A, 9A1–A2). The *2.3 Col1-Cre;Ern1* ^*fl/fl*^ *;Dspp* ^*P19L/+*^ mice showed a *Dspp* mRNA level that was comparable to *Ern1* ^*fl/fl*^ *;Dspp* ^*P19L/+*^ mice (Figures 9A, 9A1–A2). The qPCR analysis further confirmed that *Ern1* ^*fl/fl*^ *;Dspp* ^*P19L/+*^ and *2.3 Col1-Cre;Ern1* ^*fl/fl*^ *;Dspp* ^*P19L/+*^ mice showed a similar but significant and drastic decrease in *Dspp* mRNA level, compared to *Ern1* ^*fl/fl*^ control mice as well as *2.3 Col1-Cre*;*Ern1* ^*fl/fl*^ mice (Figure 10A). IHC showed that both *Ern1* ^*fl/fl*^ *;Dspp* ^*P19L/+*^ and *2.3 Col1-Cre;Ern1* ^*fl/fl*^ *;Dspp* ^*P19L/+*^ mice had increased DSP/DSPP immunostaining signals within the odontoblasts, and decreased DSP/DSPP signals in the dentin matrix, compared to *Ern1* ^*fl/fl*^ control mice (Figures 11A,A1–A2). Further, the DSP/DSPP immunostaining signals appeared to be more intense within the odontoblasts in *2.3 Col1-Cre;Ern1* ^*fl/fl*^ *;Dspp* ^*P19L/+*^ mice, compared to *Ern1* ^*fl/fl*^ *;Dspp* ^*P19L/+*^ mice (Figures 11A,A1–A2). In addition, we found that both *Ern1* ^*fl/fl*^ *;Dspp* ^*P19L/+*^ and *2.3 Col1-Cre;Ern1* ^*fl/fl*^ *;Dspp* ^*P19L/+*^ mice had a similar and substantial increase in the expression of the *Dmp1* gene, as evidenced by *in situ* hybridization (Figures 9B, 9B1–B2), qPCR (Figure 10B), and IHC results (Figures 11B,B1–B2). Taken together, the data demonstrate that the level of *Dspp* mRNA remained low after IRE1α was inactivated in the P19L-DSPP mutant mice.

## 4 Discussion

We previously reported that both *Dspp* ^*P19L/+*^ and *Dspp* ^*P19L/P19L*^ mice develop a human DGI-like phenotype (Liang et al., 2019; Liang et al., 2021). In this study, we found that IRE1α-XBP1S signaling was weakly activated in the odontoblasts in the P19L-DSPP mutant mice. We also showed that 2.3 *Col1-Cre*-mediated IRE1α inactivation caused reduced dentin formation in the wild-type mice and exacerbated the dental defects and failed to restore the reduced *Dspp* mRNA level to normal in the P19L-DSPP mutant mice. Nevertheless, the loss of IRE1α function normalized the thickened dental pulp chamber floor dentin in the P19L-DSPP mutant mice.

Several lines of evidence have supported that the mutant P19L-DSPP protein was accumulated within the ER of the odontoblasts in the P19L-DSPP mutant mice. DSPP is a secreted non-collagenous extracellular matrix protein. Previous studies show that its efficient trafficking out of the ER requires the assistance of a cargo receptor called “surfeit locus protein 4 (SURF4)” that binds to the tripeptide at the amino-terminus of the mature DSPP protein (von Marschall et al., 2012; Nam et al., 2014; Yin et al., 2018). P19 is the second amino acid residue of the amino-terminal isoleucine-proline-valine (IPV) tripeptide of mouse DSPP protein after the signal peptide is removed (Nam et al., 2014). The P19L substitution would presumably affect the interaction of the mutant P19L-DSPP with SURF4, resulting in its accumulation within the ER (von Marschall et al., 2012; Nam et al., 2014; Yin et al., 2018). Indeed, we have shown that there was an accumulation of the mutant P19L-DSPP protein within the odontoblasts in *Dspp* ^*P19L/+*^ and *Dspp* ^*P19L/P19L*^ mice (Liang et al., 2019). We have also demonstrated that the mutant P19L-DSPP protein was accumulated within the ER in the expressing cells (Liang et al., 2019; Liang et al., 2023). In this study, our Western-blotting and Stains-all staining analyses of the total proteins extracted from the dental pulps/odontoblasts and dentin matrices further supported that the mutant P19L-DSPP protein was not efficiently secreted out of the odontoblasts, resulting in its intracellular accumulation. An accumulation of the mutant P19L-DSPP protein in the ER may disrupt ER homeostasis and cause ER stress.

Therefore, we analyzed the IRE1α branch of the UPR to determine if it was abnormally activated by the intracellularly accumulated mutant P19L-DSPP protein. IRE1α is the most evolutionally conserved ER stress sensor (Mori et al., 1993; Cox and Walter, 1996; Tirasophon et al., 1998; Wang et al., 1998; Koizumi et al., 2001). We showed that the immunostaining signals for phosphorylated IRE1α were strongly detected in the floor-forming odontoblasts and other dental pulp cells of 3-week-old *Dspp* ^*P19L/+*^ and *Dspp* ^*P19L/P19L*^ mice, compared to the *Dspp* ^*+/+*^ control mice. Moreover, there was a dramatic increase in the immunostaining signals for total XBP1, including both XBP1U and XBP1S, in the odontoblasts as well as other dental pulp cells in *Dspp* ^*P19L/+*^ and *Dspp* ^*P19L/P19L*^ mice. However, the XBP1S-specific immunostaining signals were very weak in all three groups of mice but appeared to be slightly stronger in *Dspp* ^*P19L/+*^ and *Dspp* ^*P19L/P19L*^ mice than in *Dspp* ^*+/+*^ control mice. Further analysis of *Xbp1* mRNA levels using a combination of RT-PCR and enzymatic digestion approaches showed that the increase in *Xbp1s* mRNA was quite small in the P19L-DSPP mutant mice. These findings indicate that the IRE1α-XBP1S signaling was minimally activated in *Dspp* ^*P19L/+*^ and *Dspp* ^*P19L/P19L*^ mice at the age of 3 weeks.

To further determine the roles of IRE1α in normal and abnormal dentinogenesis, we generated *2.3 Col1-Cre*;*Ern1* ^*fl/fl*^ mice and compound *2.3 Col1-Cre;Ern1* ^*fl/fl*^ *;Dspp* ^*P19L/+*^ mice with the *Ern1* gene (encoding IRE1α) deleted in the odontoblasts in the wild-type and P19L-DSPP mutant mouse genetic background, respectively. We found that *2.3 Col1-Cre*;*Ern1* ^*fl/fl*^ mice exhibited a significant reduction in total dentin/cementum volume, but not a significant decrease in dentin/cementum density, compared to *Ern1* ^*fl/fl*^ control mice, at the two ages examined. The reduced dentin formation may result from the loss of the IRE1α-XBP1S signaling in the odontoblasts in *2.3 Col1-Cre*;*Ern1* ^*fl/fl*^ mice as it has been previously shown that the IRE1α-XBP1S signaling is essential for osteoblast differentiation and bone formation (Tohmonda et al., 2011). The *Ern1* ^*fl/fl*^ *;Dspp* ^*P19L/+*^ mice showed a significant decrease in the thickness of dental pulp chamber roof dentin, but a significant increase in the thickness of pulp chamber floor dentin, accompanied by a thinner roof predentin as well as shorter and irregularly arranged odontoblasts, a dental phenotype that is similar to that of *Dspp* ^*P19L/+*^ mice (Liang et al., 2019). The *2.3 Col1-Cre;Ern1* ^*fl/fl*^ *;Dspp* ^*P19L/+*^ mice developed more severe dental defects with regards to the dental pulp chamber roof dentin thickness, dentin/cementum volume, dental pulp chamber size, and odontoblast morphology, compared to *Ern1* ^*fl/fl*^ *;Dspp* ^*P19L/+*^ mice. Overall, the data demonstrate that IRE1α is crucial for odontoblast function and dentin formation in the wild-type mice as well as in the P19L-DSPP mutant mice.

Moreover, it is important to note that loss of IRE1α function produced different effects on the pulp chamber roof and floor dentin formation in different genetic background. In *2.3 Col1-Cre*;*Ern1* ^*fl/fl*^ mice, IRE1α inactivation reduced the thickness of the dental pulp chamber roof dentin, but it increased the thickness of pulp chamber floor dentin, though it was not significant when compared to *Ern1* ^*fl/fl*^ control mice, at the age of 3 weeks. However, the thickness of the pulp chamber floor dentin in *2.3 Col1-Cre*;*Ern1* ^*fl/fl*^ mice became comparable to that in *Ern1* ^*fl/fl*^ mice by the age of 7 weeks. In contrast, in *2.3 Col1-Cre;Ern1* ^*fl/fl*^ *;Dspp* ^*P19L/+*^ mice, IRE1α deficiency not only decreased the thickness of the pulp chamber roof dentin at the two ages examined, but it also significantly reduced the thickness of the pulp chamber floor dentin by the age of 7 weeks, compared to those in *Ern1* ^*fl/fl*^ *;Dspp* ^*P19L/+*^ mice. It is of note that the thickness of the dental pulp chamber floor dentin in *2.3 Col1-Cre;Ern1* ^*fl/fl*^ *;Dspp* ^*P19L/+*^ mice was reduced to a level that is close to that in age-matched *Ern1* ^*fl/fl*^ control mice. Along these lines, the immunostaining signals for phosphorylated IRE1α were much stronger in the floor-forming odontoblasts but were barely detectable in the roof-forming odontoblasts of 3-week-old *Dspp* ^*P19L/+*^ and *Dspp* ^*P19L/P19L*^ mice. These findings support that enhanced IRE1α function may account for the increased formation of dental pulp chamber floor dentin in the P19L-DSPP mutant mice, indicating a context-dependent pathogenic role of IRE1α in tooth development and disease.

The different effects of the mutant P19L-DSPP and the loss of IRE1α function on the roof and floor dentin formation may result from the inherent differences between the roof- and floor-forming odontoblasts. Developmentally, the roof-forming odontoblasts are derived from the mesenchymal cells as a result of the reciprocal interactions between the enamel organ and dental mesenchyme during tooth development (Thesleff and Sharpe, 1997). However, the floor-forming odontoblasts are differentiated from the mesenchymal cells induced by the Hertwig’s epithelial root sheath (Thomas and Kollar, 1989; Li et al., 2017). Once differentiated, the roof-forming odontoblasts assume a columnar shape whereas the floor-forming odontoblasts appear cuboidal (Thomas, 1995). Moreover, the roof- and floor-forming odontoblasts show differences in the quantity and quality of the genes they express, including those genes encoding extracellular matrix proteins (Steinfort et al., 1989; Andujar et al., 1991; Lavicky et al., 2022). Consistently, our current and previous studies have demonstrated that the roof-forming odontoblasts express a higher level of DSPP than the floor-forming odontoblasts in both wild-type and P19L-DSPP mutant mice (Liang et al., 2019). Thereby, it is possible that the difference in the levels of the mutant P19L-DSPP along with the inherent differences between the two populations of odontoblasts leads to the differential activation of IRE1α in the roof- and floor-forming odontoblasts. Similar mechanism may also account for the differential effects of IRE1α deletion on the roof and floor dentin formation in different genetic background. IRE1α inactivation results in reduced overall dentin formation, accompanied by decreased *Dspp* expression in *2.3 Col1-Cre*;*Ern1* ^*fl/fl*^ mice. Therefore, it is putative that the elevated IRE1α-XBP1S signaling may account for the increased floor dentin formation through upregulating *Dspp* expression in the floor-forming odontoblasts in the P19L-DSPP mutant mice. However, how the mutant P19L-DSPP and IRE1α ablation cause different effects on the roof- and floor-forming odontoblasts remains to be further investigated.

In addition to the IRE1α-XBP1S signaling, activated IRE1α cleaves other ER-localized mRNAs, resulting in their degradation through regulated IRE1-dependent decay (RIDD), thereby reducing the ER load under stressed conditions (Hollien and Weissman, 2006; Han et al., 2009; Hollien et al., 2009; Nakamura et al., 2011; Gaddam et al., 2013; Moore and Hollien, 2015). Our previous (Liang et al., 2019) as well as current studies have demonstrated that the level of *Dspp* mRNAs was dramatically reduced in the odontoblasts in *Dspp* ^*P19L/+*^, and *Dspp* ^*P19L/P19L*^ mice, suggesting that RIDD might occur in the odontoblasts in the P19L-DSPP mutant mice. However, we found that inactivation of IRE1α did not alter the levels of *Dspp* mRNAs in the P19L-DSPP mutant mice, as compound *2.3 Col1-Cre;Ern1* ^*fl/fl*^ *;Dspp* ^*P19L/+*^ mice had a level of *Dspp* mRNAs that was comparable to *Ern1* ^*fl/fl*^ *;Dspp* ^*P19L/+*^ mice. Additionally, RIDD requires a consensus sequence (CUGCAG) along with a secondary stem-loop structure on the targeted mRNAs (Oikawa et al., 2010; Hur et al., 2012; Moore and Hollien, 2015). Yet, such consensus sequence/structure was not found when mouse *Dspp* mRNA sequence (GenBank Accession: NM_010080) was examined. Taken together, our findings rule out the possibility that RIDD contributes to the decrease in *Dspp* mRNA levels in the P19L-DSPP mutant mice. Further studies are warranted to understand how the level of *Dspp* mRNAs is reduced in the P19L-DSPP mutant mice.

In contrast to DSPP, our current and previous studies consistently show that DMP1 is dramatically elevated at both mRNA and protein levels in the P19L-DSPP mutant mice (Liang et al., 2019). Both DMP1 and DSPP are members of the small integrin-binding ligand N-linked glycoprotein family (Fisher et al., 2001), and are essential for dentin formation (Sreenath et al., 2003; Ye et al., 2004). In addition to its role as a hydroxyapatite nucleator in the extracellular matrix (He et al., 2003), DMP1 has been shown to enter the nucleus and function as a transcription factor that regulates osteoblast differentiation (Narayanan et al., 2003). It has also been shown that DMP1 upregulates the expression of *Dspp* during odontoblast differentiation (Narayanan et al., 2006). Consistently, deletion of *Dmp1* results in a decrease in the level of DSPP, and *Dmp1*-null mice manifest a tooth phenotype similar to that of *Dspp*-null mice (Sreenath et al., 2003; Ye et al., 2004). Further, transgenic expression of DSPP rescues the tooth defects of *Dmp1*-null mice, demonstrating that DMP1 regulates dentin formation through DSPP (Gibson et al., 2013). Altogether, these findings indicate that increased DMP1 expression may serve as a compensatory feedback mechanism in odontoblasts. In response to reduced *Dspp* expression, *Dmp1* is upregulated, with the potential to increase *Dspp* expression in the P19L-DSPP mutant mice.

In summary, our current study underscores the critical roles of IRE1α in odontoblast function and dentin formation. Moreover, we corroborate the pathogenic roles of enhanced IRE1α activity in the mutant P19L-DSPP mice, particularly in the increased formation of dental pulp chamber floor dentin. In the meantime, we exclude the involvement of IRE1α in the degradation of *Dspp* mRNA. Therefore, further studies are still needed to completely understand the molecular pathogenesis of dentinogenesis imperfecta associated with *DSPP* mutations in order to develop an effective and preventative therapeutic strategy for clinical management of DGI patients in the future.

## Data Availability

The original contributions presented in the study are included in the article/supplementary material, further inquiries can be directed to the corresponding author.
